# Toxins in Drug Discovery and Pharmacology

**DOI:** 10.3390/toxins10030126

**Published:** 2018-03-16

**Authors:** Steve Peigneur, Jan Tytgat

**Affiliations:** Toxicology and Pharmacology, University of Leuven (KU Leuven), Campus Gasthuisberg, P.O. Box 922, Herestraat 49, 3000 Leuven, Belgium; jan.tytgat@pharm.kuleuven.be

Venoms from marine and terrestrial animals (cone snails, scorpions, spiders, snakes, centipedes, cnidarian, etc.) can be seen as an untapped cocktail of biologically active compounds, being increasingly recognized as a new emerging source of peptide-based therapeutics. Venomous animals are considered specialized predators that have evolved the most sophisticated peptide chemistry and neuropharmacology for their own biological purposes by producing venoms that contain myriads of toxins with an amazing structural and functional diversity. These neurotoxins have been shown to be highly selective and potent ligands for a wide range of ion channels and receptors. Therefore, they represent interesting lead compounds for the development of novel medicines, for example, analgesics, anti-cancer drugs, and drugs for neurological disorders. A successful example of venom-derived peptides used is the drug Prialt^®^ or Ziconotide, which is a synthetic version of a peptide that is found in the venom of a marine snail, *Conus magus*. It is a 25-amino acid residue peptide blocking N-type voltage-gated calcium channels. Its FDA definition reads that ziconotide is intended for the treatment of chronic severe pain in patients that are intolerant or refractory to systemic analgesics or intrathecal morphine [1]. Characterization of small, bradykinin potentiating peptides from the venom of the South American snake *Bothrops jararaca* led to the development of Captopril, even prior to Prialt^®^. Captopril, and its follow-up compounds, are widely used as inhibitors of angiotensin converting enzyme (ACE) [2,3,4]. Another example is Exenatide^®^, which is a synthetic version of a hormone called exendin-4 isolated from the saliva of the glia monster [5]. Exendin-4 is a glucagon-like peptide-1 receptor agonist and hereby finds its use in the treatment of type 2 diabetes [6]. Exenatide^®^ has been approved by the FDA since 2005 [7]. 

In this special issue, “Toxins in Drug Discovery and Pharmacology”, we have attempted to provide the reader with a comprehensive overview of new toxins and toxin-inspired leads. This issue focuses on the mechanism of action, structure–function, and the evolution of pharmacologically interesting venom components, including, but not limited to, recent developments relating to the emergence of venoms as an underutilized source of highly evolved bioactive peptides with clinical potential. The following is a short synopsis of the six reviews and 15 research papers that constitute this special issue.

Agwa and colleagues [8] have explored the pharmaceutical potential of spider venoms. Their work shows that spider-derived gating modifier toxins are undeniably among nature’s more interesting pharmacological probes in the study of voltage-gated ion channels. The current work has provided additional insight into the potential of these ICK peptides as templates for drugs designed to target ailments linked to the voltage-gated ion channels. Freitas et al. [9] also focused on spider venoms for the discovery of novel lead compounds with potential interesting pharmaceutical properties. PnPP-19 is a toxin-derived peptide from *Phoneutria nigriventer* that activates μ-opioid receptors without inducing β-arrestin2 recruitment. PnPP-19 is the first spider toxin derivative that, among opioid receptors, selectively activates μ-opioid receptors. The lack of β-arrestin2 recruitment highlights its potential for the design of new improved opioid agonists. 

Dong et al. [10] describe a high-throughput way to discover AMPs from fish gastrointestinal microbiota, which can be developed as alternative pathogen antagonists or toxins for micro-ecologics or probiotic supplements.

The anti-cancer properties of snake venom have been investigated by Osipov et al. by verifying the anti-tumor effects of nerve growth factor from cobra venom. This work suggests that the antitumor effect of nerve growth factor in vivo depends critically on the normal status of the immune system. The nerve growth factor antitumor mechanism may cause an increase in lymphocytic infiltration in the tumor, a rise in the levels of IL-1β and TNF-α in the serum of tumor-bearing mice, and an increase in aerobic glycolysis [11]. Sales et al. [12] questioned whether inhibitors of snake venom phospholipases A2 can lead to new insights into anti-inflammatory therapy in humans. This work reports a proof-of-principle study that snake venom toxins, more specifically snake venom phospholipases A2, can be used as tools for studies in human phospholipases A2, taking care in choosing the more specifically snake venom phospholipases A2. Azemiopsin is a linear peptide from viper venom, and it is a selective inhibitor of nicotinic acetylcholine receptors. Azemiopsin has good drug-like properties for the application as local muscle relaxant [13].

Another source for venom-based drug discovery is bee venom. Bee venom given subcutaneously attenuates allodynia in mice models of CPIP without notable adverse effects. The anti-allodynic effects were closely associated with a significant decrease in NK-1 receptor expression in DRG. These findings suggest that repetitive bee venom therapy could be a useful therapeutic modality for the treatment of complex regional pain syndrome type I [14]. Shin and colleagues [15] report that melittin and apamin can inhibit the fungi-induced production of chemical mediators and ECM from nasal fibroblasts. This study suggests the possible role of melittin and apamin in the treatment of fungi-induced airway inflammatory diseases. Another study with bee venom showed that bee venom acupuncture has potent suppressive effects against paclitaxel-induced neuropathic pain, which is mediated by spinal α2-adrenergic receptor activity [16]. Bee venom may be a useful preventive and therapeutic agent in the treatment of obesity. It was found that bee venom mediates anti-obesity effects by suppressing obesity-related transcription factors [17].

Lepiarczyk and colleagues [18] describe a first study to suggest that both resiniferatoxin and tetrodotoxin can modify the number of noradrenergic and cholinergic NF supplying the porcine urinary bladder.

The mastoparan V1 is a mastoparan from the venom of the social wasp *Vespula vulgaris* with potent antimicrobial activity against Salmonella infection. However, there exist some limits for its practical application due to the loss of its activity in an increased bacterial density and the difficulty of its efficient production. Ha et al. [19] modulated successfully the antimicrobial activity of synthetic mastoparan V1 against an increased Salmonella population using protease inhibitors. Furthermore, they developed an *Escherichia coli* secretion system efficiently producing active mastoparan V1.

Short toxin-like proteins from insects are often overlooked in drug discovery. The study performed by Linial et al. [20] indicates dozens of new candidates for peptide-based therapy and their potential for drug design is discussed. It was concluded that the overlooked endogenous toxin-like proteins from insects characterized by the structural stability and enhanced specificity are attractive templates for drug design.

Two dipeptides with anticoagulant activity have been isolated from scorpion venom [21]. This study shows for the first time the ability of short venom peptides to slow down blood coagulation. Using molecular dynamics simulation of complexes between scorpion toxins and the Kv1.2 channel resulted in the identification of hydrophobic patches, hydrogen-bonds, and salt bridges as the three essential forces mediating the interactions between this channel and the toxins. This discovery might help design highly selective Kv1.2-channel inhibitors [22].

Park and Park [23] provided a comprehensive review to summarize the experimental and clinical evidence of the mechanism by which Botulinum toxin acts on various types of neuropathic pain and describe why Botulinum toxin is a successful example of toxin-based drug discovery. Botulinum toxin has been used for approximately 40 years for treatment of excessive muscle stiffness, spasticity, dystonia, and various types of neuropathic pain.

Royal and Motoba [24] review the possible mechanisms behind the cholera toxin B subunit anti-inflammatory activity and discuss how the protein could impact mucosal inflammatory disease treatment.

The anti-metastatic mechanisms of snake toxins can be divided into three molecular targets. These are the inhibition of extracellular matrix components-dependent adhesion and migration, the inhibition of the epithelial–mesenchymal transition and the inhibition of the migration by alterations in the actin/cytoskeleton network. The molecular mechanisms in which snake toxins target metastasis are reviewed by Urra and Araya-Maturana [25].

The genus *Conus* has become an important genetic resource for conotoxin identification and drug development. The many challenges of drug discovery from cone snail venom are reviewed by Gao et al. [26].

Animal toxins are valuable tools to study ion channels such as TRPV1. A comprehensive summary of the advancements made in TRPV1 research in recent years by employing venom-derived peptide toxins is provided by Geron and colleagues [27]. In this review, the authors describe for each toxin its functional aspects, behavioral effects, and structural features, all of which have contributed to our current knowledge of TRPV1.
